# Basal Cell Carcinoma and Sebaceoma Within Nevus Sebaceous of the Scalp

**DOI:** 10.7759/cureus.18632

**Published:** 2021-10-09

**Authors:** Katherine David, Mandy Alhajj, Jeffrey Parks, Rabail Aslam, Jessica Sigel

**Affiliations:** 1 Dermatology, Lake Erie College of Osteopathic Medicine, Greensburg, USA; 2 Dermatology, University Hospitals Cleveland Medical Center, Cleveland, USA; 3 Surgery, UH Regional Hospitals, Richmond Heights, USA; 4 Pathology, University Hospitals Cleveland Medical Center, Cleveland, USA

**Keywords:** scalp, muir-torre syndrome, basal cell carcinoma, nevus sebaceous, sebaceoma

## Abstract

This case describes the occurrence of basal cell carcinoma (BCC) and sebaceoma within a nevus sebaceous (NV), which has not yet been previously reported. This is significant to dermatologists as it emphasizes the importance of close monitoring of benign sebaceous nevi in the event that malignant transformation occurs, although such occurrences are rare. Prompt consideration for prophylactic excision of NS is warranted prior to malignant transformation.

## Introduction

Nevus sebaceous (NS) is a rare hamartoma with an incidence of 0.05%-1% diagnosed in childhood [1]. Malignant neoplasms arise in less than 3% of cases, almost exclusively in adults [1]. In this article, we discuss the case of a 65-year-old female with no history of Muir-Torre disease who was found to have both basal cell carcinoma (BCC) and sebaceoma within a long-standing NS of the scalp requiring extensive surgical intervention.

## Case presentation

**Figure 1:**
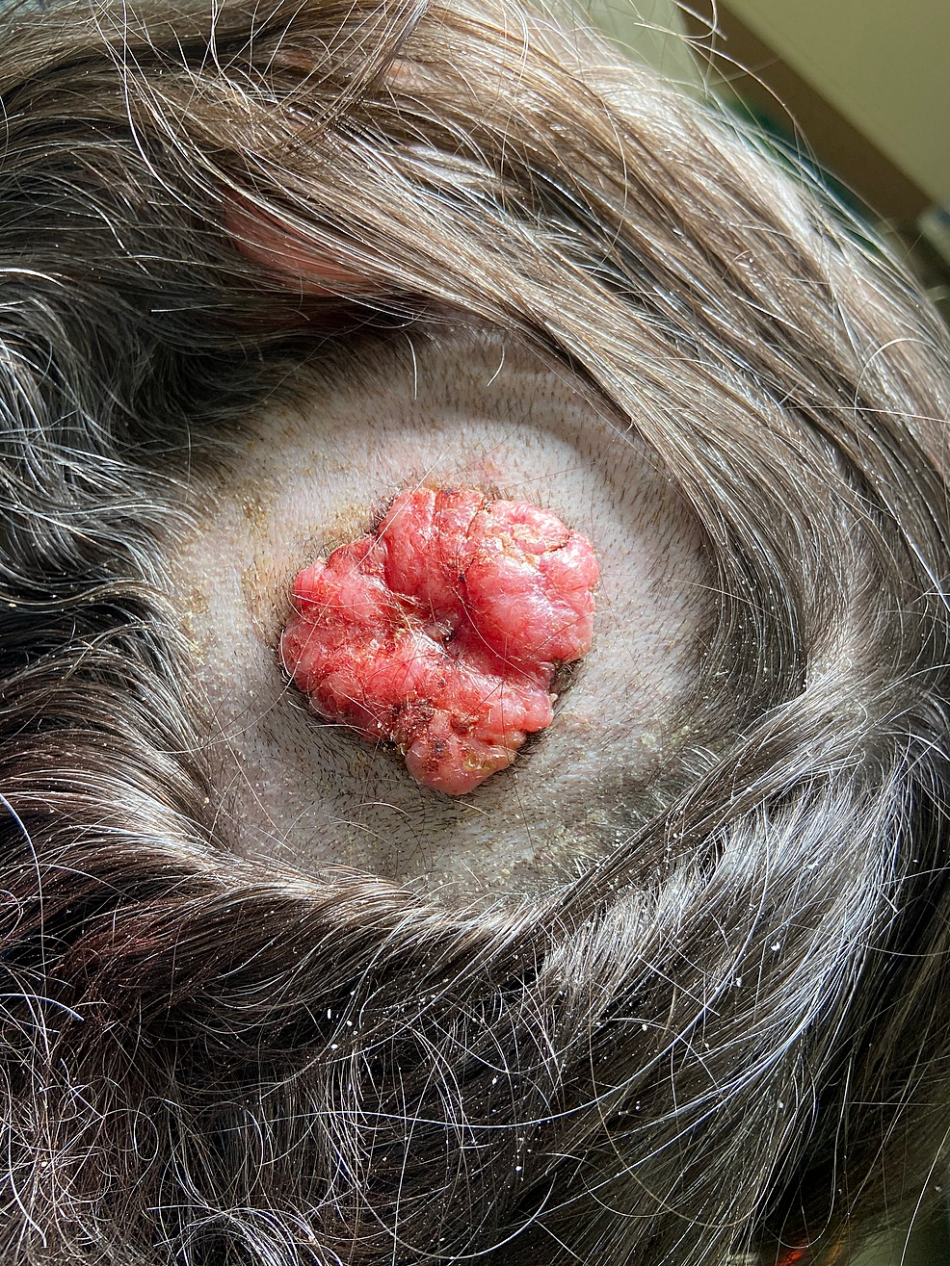
Intraoperative nevus sebaceous

**Figure 2:**
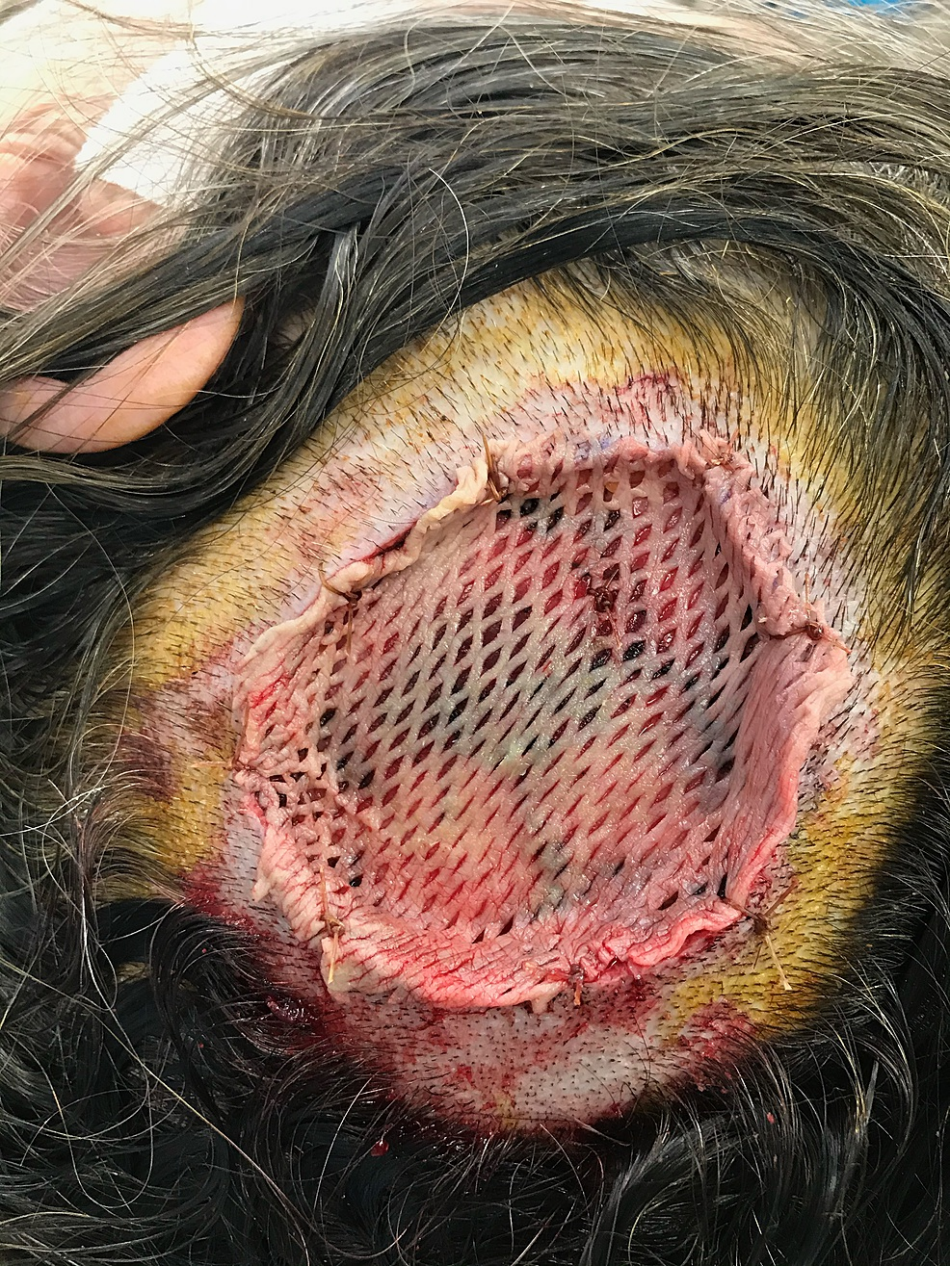
Intraoperative split-thickness skin graft overlying the left parietal-temporal region following wide en bloc excision

**Figure 3:**
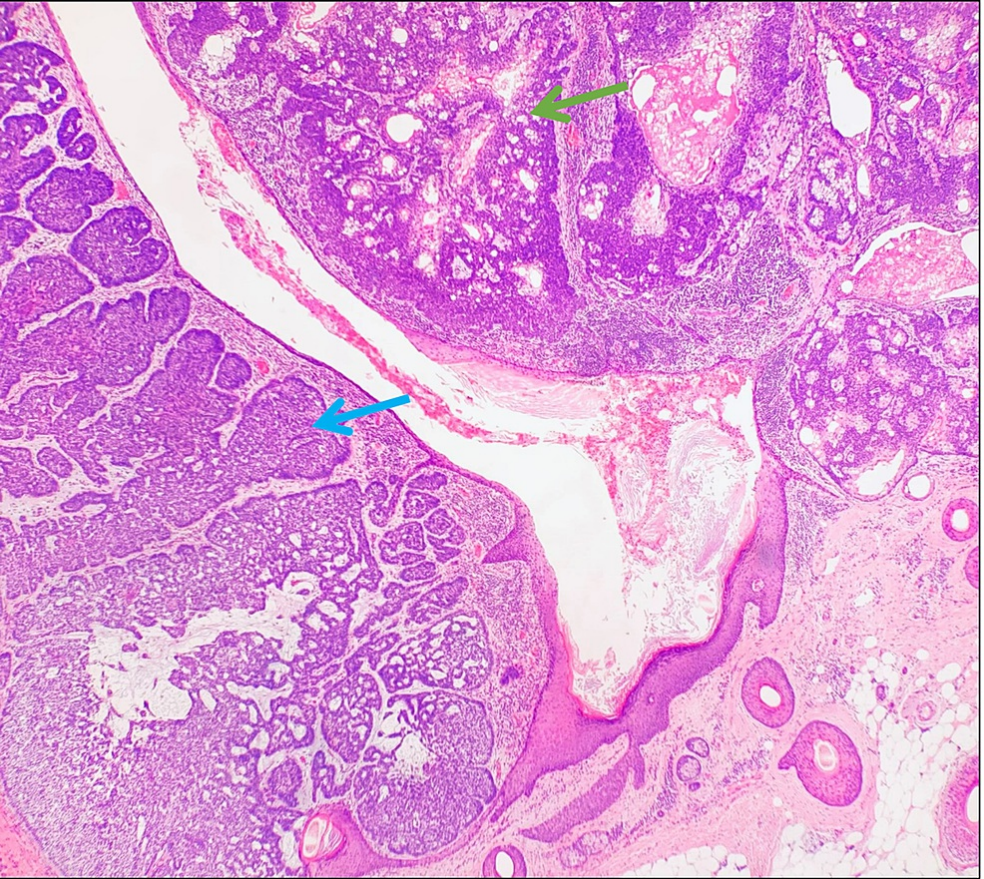
Low-power image of basal cell carcinoma (blue arrow) and sebaceoma (green arrow) on the right existing concurrently in the lesion

**Figure 4:**
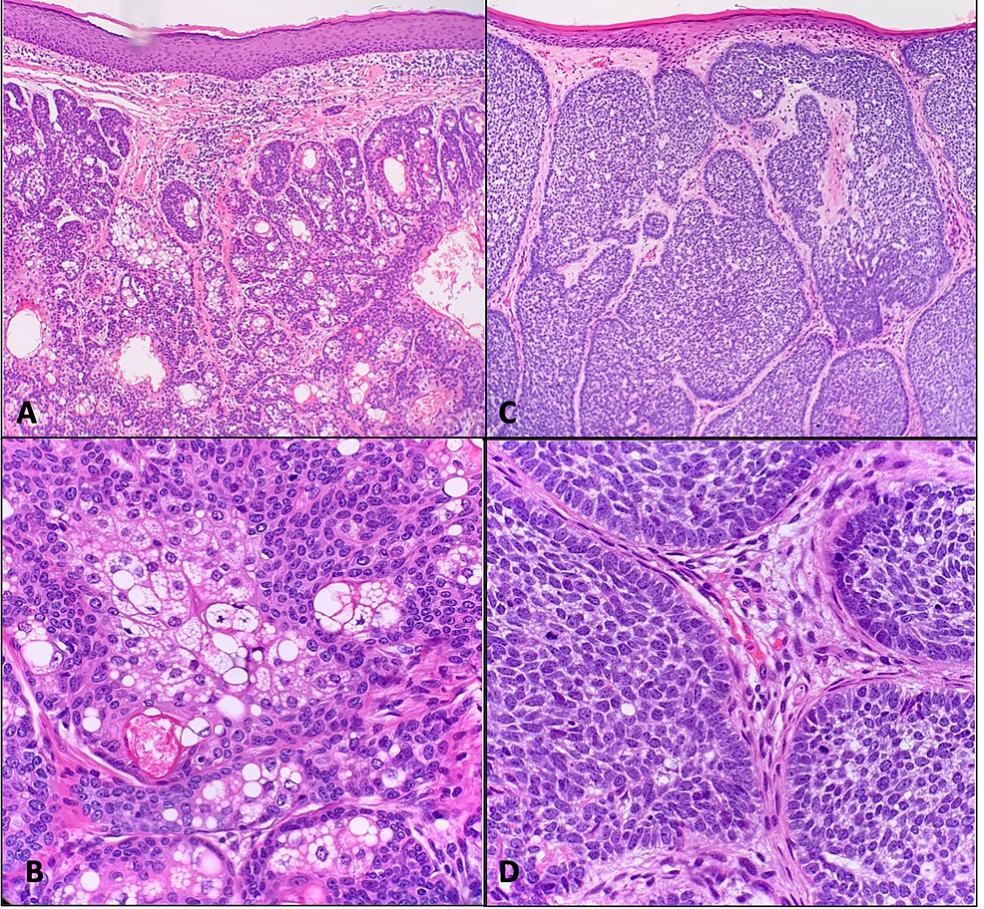
A. Low-magnification image of intradermal sebaceoma. B. High-magnification image of sebaceoma demonstrating multivacoulated sebocytes. C. Nodular basal cell carcinoma (BCC) with peripheral palisading pattern. D. High-power view of BCC demonstrating peripheral palisading.

A 65-year-old female presented to the surgery clinic with a six-week history of a pruritic, bleeding scalp mass in the left parietal-temporal region. The lesion had been asymptomatic since childhood but had grown over this six-week period. Examination of the scalp revealed a raised, fungated lesion measuring 4.7 x 4.0 cm with overlying alopecia secondary to the lesion (Figure 1). The irregular mass was erythematous with telangiectasias and several areas of punctate bleeding and erosion. The mass was subsequently resected via wide en bloc excision with split-thickness skin graft closing utilizing tissue from the patient's left medial thigh (Figure 2). Biopsy revealed a large basal cell carcinoma with predominantly nodular and focal infiltrating features occurring concurrently with an adjacent sebaceous neoplasm (Figures 3 and 4). The latter was comprised of well-circumscribed nodules of basaloid cells with mild to focal moderate cytologic atypia and frequent mitoses with an absence of peripheral palisading and retraction artifact favoring a sebaceoma.

 

## Discussion

NS affect both surface epithelial and adnexal components of the skin and present as flat, hairless, and yellow-orange in color, with overlying alopecia [1]. These congenital lesions are typically benign, susceptible to hormonal changes, and undergo proliferation of the sebaceous and apocrine glands and epidermal hyperplasia during puberty [1]. If neoplastic changes occur, the most frequent tumors seen are trichoblastoma (34.7%), syringocystadenoma papilliferum (SCAP) (24.7%), apocrine adenoma (10%), and trichilemmoma (5.3%) [2]. BCC has been found to develop within NS at an incidence of approximately 1% [1]. 

While studies have been limited on NS and subsequent malignancy, a heterozygous mutation most often found in several independent NS tissues has been identified as either *HRAS* or *KRAS* mutations, confirming a strong correlation between activating *RAS* mutations and NS [3].

It is uncommon for multiple tumor types to develop within a single NS, although multiple types of tumors can occur within NS lesions. BCC alone within NS has been noted in some cases predominantly affecting the trunk and scalp [4,5]. BCC and sebaceoma have rarely been documented to occur concurrently within an NS. The limited number of reported cases show clinical differences and present a challenge in establishing clinical guidelines. For example, the presence of sebaceoma has been reported to be associated with Muir-Torre syndrome, which was diagnostically excluded in our patient [6]. 

Treatment of NS typically consists of local excision secondary to the risk of malignant transformation or for cosmetic purposes [1]. There is no consensus within the literature on the management of NS, and strict guidelines have not been documented. Some clinicians recommend close monitoring of the lesions, while others encourage early prophylactic excision. Our case illustrates the necessity of close observation of NS due to the risk of malignant transformation and is unique in that both BCC and sebaceoma, which is a rare entity of its own, presented within an NS. Ultimately, the time of surgical excision should be determined on a case-by-case basis with the goal of improving the patient's quality of life.

## Conclusions

This case discusses the malignant transformation of a nevus sebaceous. The patient presented to surgery with a fungated lesion that grew increasingly pruritic and friable over a six-week period. Excision and histologic examination of the lesion revealed basal cell carcinoma and sebaceoma embedded in the nevus sebaceous. This case is significant due to the rarity of sebaceomas and the presence of both BCC and sebaceoma within a single nevus sebaceous. Management of NS remains somewhat controversial as malignant transformation remains low risk. Some studies suggest conservative management through observation, while others prefer early excision. Excision of the lesions during childhood or adolescence is not required but may be beneficial as margins around the NS lesions can remain smaller than in the case of malignant transformation in adulthood. In the event of malignant change, although rare, more extensive procedures may be required, as seen in our case. This report serves as a reminder of the importance of close monitoring of NS and the recommendation of prophylactic excision of an evolving nevus sebaceous to yield optimal results.
